# Supplementary figures and images for: Correlating fluorescence microscopy, optical and magnetic tweezers to study single chiral biopolymers such as DNA
Source: Nat Commun. 2024 Mar 29;15:2748. doi: 10.1038/s41467-024-47126-6 (PMC10980717; doi:10.1038/s41467-024-47126-6)

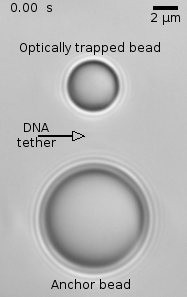

Supplement: Supplementary file 3 — Supplementary Movie 2 [file 41467_2024_47126_MOESM3_ESM.gif]

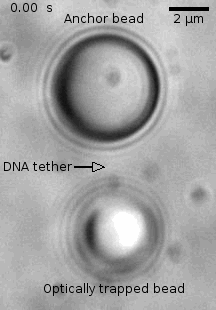

Supplement: Supplementary file 4 — Supplementary Movie 3 [file 41467_2024_47126_MOESM4_ESM.gif]

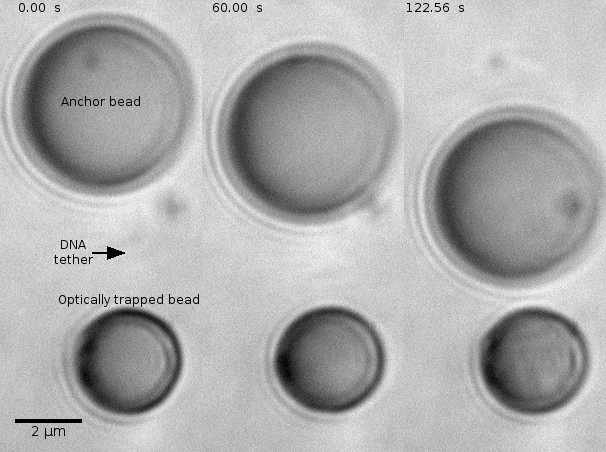

Supplement: Supplementary file 5 — Supplementary Movie 4 [file 41467_2024_47126_MOESM5_ESM.gif]

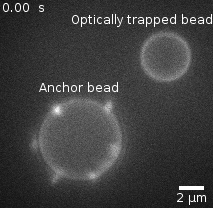

Supplement: Supplementary file 6 — Supplementary Movie 5 [file 41467_2024_47126_MOESM6_ESM.gif]

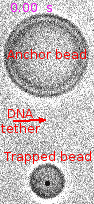

Supplement: Supplementary file 7 — Supplementary Movie 6 [file 41467_2024_47126_MOESM7_ESM.gif]

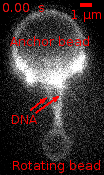

Supplement: Supplementary file 8 — Supplementary Movie 7 [file 41467_2024_47126_MOESM8_ESM.gif]

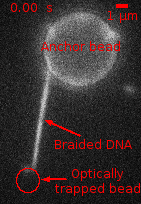

Supplement: Supplementary file 9 — Supplementary Movie 8 [file 41467_2024_47126_MOESM9_ESM.gif]

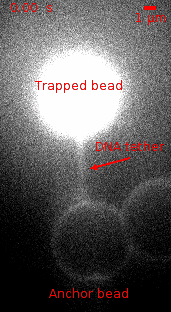

Supplement: Supplementary file 10 — Supplementary Movie 9 [file 41467_2024_47126_MOESM10_ESM.gif]

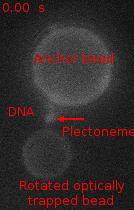

Supplement: Supplementary file 11 — Supplementary Movie 10 [file 41467_2024_47126_MOESM11_ESM.gif]

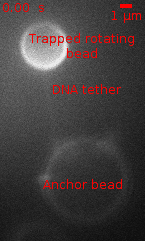

Supplement: Supplementary file 12 — Supplementary Movie 11 [file 41467_2024_47126_MOESM12_ESM.gif]

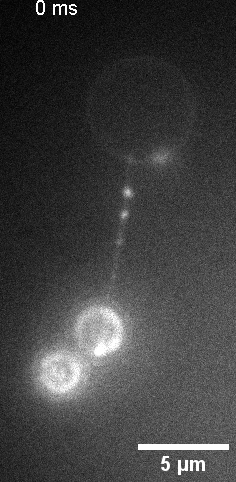

Supplement: Supplementary file 13 — Supplementary Movie 12 [file 41467_2024_47126_MOESM13_ESM.gif]

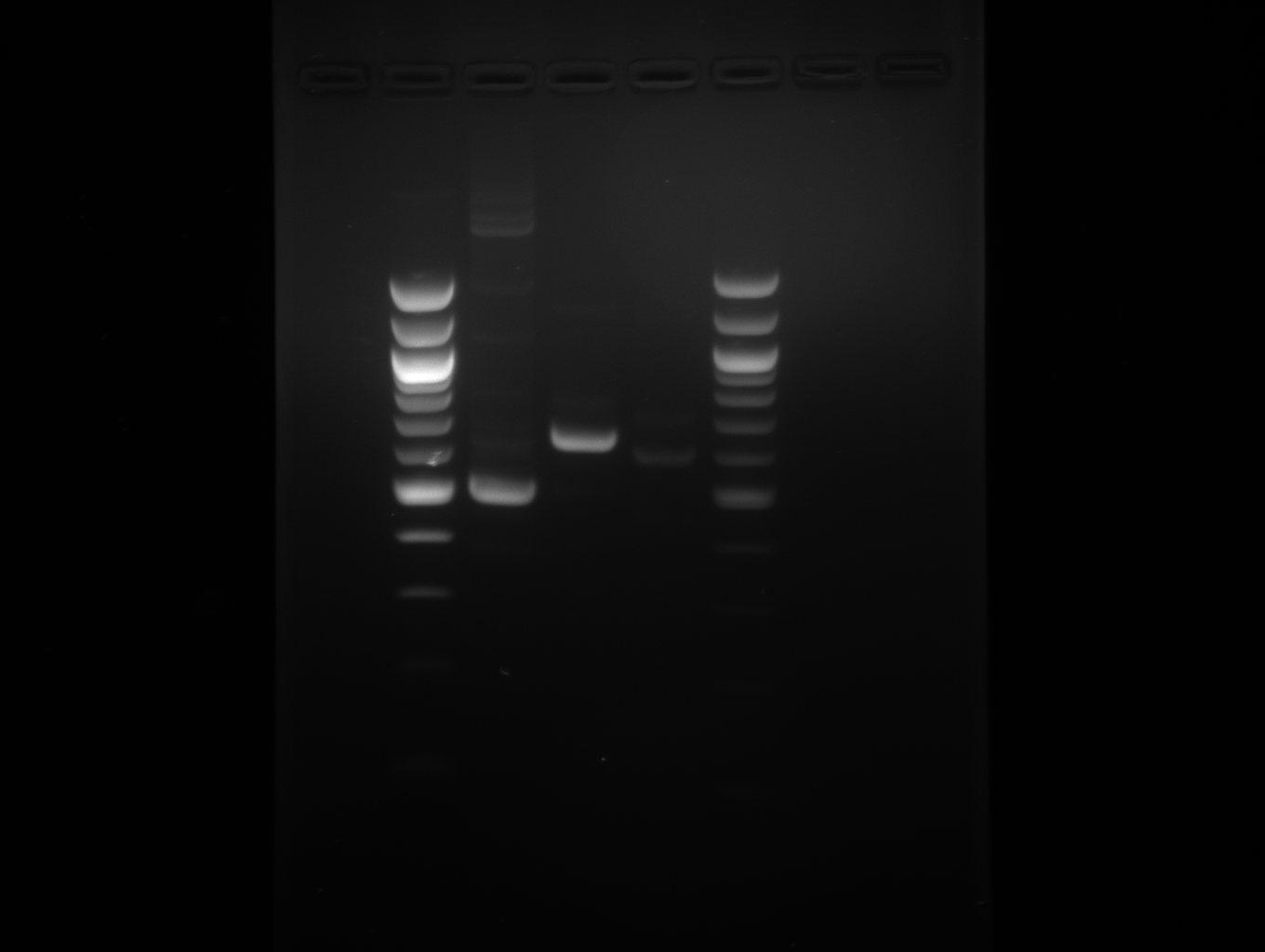

Supplement: Supplementary file 21 — Source Data [file 41467_2024_47126_MOESM21_ESM.zip › source data_final/suppl figure 9b/210428 handles.tif]
