# Supplementary material for: Correlating fluorescence microscopy, optical and magnetic tweezers to study single chiral biopolymers such as DNA
Source: Nat Commun. 2024 Mar 29;15:2748. doi: 10.1038/s41467-024-47126-6 (PMC10980717; doi:10.1038/s41467-024-47126-6)
Supplement: Supplementary file 18 — Reporting Summary [file 41467_2024_47126_MOESM18_ESM.pdf]

nature portfolio

Corresponding author(s): Mark Leake

Last updated by author(s): Mar 5, 2024

Reporting Summary

Nature Portfolio wishes to improve the reproducibility of the work that we publish. This form provides structure for consistency and transparency in reporting. For further information on Nature Portfolio policies, see our Editorial Policies and the Editorial Policy Checklist.

Statistics

For all statistical analyses, confirm that the following items are present in the figure legend, table legend, main text, or Methods section.

n/a	Confirmed	
☐	☒	The exact sample size (*n*) for each experimental group/condition, given as a discrete number and unit of measurement
☐	☒	A statement on whether measurements were taken from distinct samples or whether the same sample was measured repeatedly
☐	☒	The statistical test(s) used AND whether they are one- or two-sided<br>*Only common tests should be described solely by name; describe more complex techniques in the Methods section.*
☐	☒	A description of all covariates tested
☐	☒	A description of any assumptions or corrections, such as tests of normality and adjustment for multiple comparisons
☐	☒	A full description of the statistical parameters including central tendency (e.g. means) or other basic estimates (e.g. regression coefficient) AND variation (e.g. standard deviation) or associated estimates of uncertainty (e.g. confidence intervals)
☐	☒	For null hypothesis testing, the test statistic (e.g. *F*, *t*, *r*) with confidence intervals, effect sizes, degrees of freedom and *P* value noted<br>*Give P values as exact values whenever suitable.*
☒	☐	For Bayesian analysis, information on the choice of priors and Markov chain Monte Carlo settings
☒	☐	For hierarchical and complex designs, identification of the appropriate level for tests and full reporting of outcomes
☒	☐	Estimates of effect sizes (e.g. Cohen's *d*, Pearson's *r*), indicating how they were calculated

Our web collection on statistics for biologists contains articles on many of the points above.

Software and code

Policy information about availability of computer code

Data collection	All code used for instrument design, instrument control, data acquisition, data processing, analysis and figure generation directly relies on the following packages: Matlab, LabVIEW, Mathematica, Autodesk Inventor, Jupyter, Matplotlib, NumPy, Pandas, SciPy. Source code for CombiTweez is available on GitHub (https://github.com/york-biophysics) and is provided under the Creative Commons Attribution-NonCommercial-ShareAlike 4.0 International License (CC-BY-NC-SA; https://creativecommons.org/licenses/by-nc-sa/4.0/). We have also created a unique DOI for this code resource at https://zenodo.org/doi/10.5281/zenodo.10779228 (2024) and added this as a reference into the methods section of manuscript.
Data analysis	All code used for instrument design, instrument control, data acquisition, data processing, analysis and figure generation directly relies on the following packages: Matlab, LabVIEW, Mathematica, Autodesk Inventor, Jupyter, Matplotlib, NumPy, Pandas, SciPy. Source code for CombiTweez is available on GitHub (https://github.com/york-biophysics) and is provided under the Creative Commons Attribution-NonCommercial-ShareAlike 4.0 International License (CC-BY-NC-SA; https://creativecommons.org/licenses/by-nc-sa/4.0/).

For manuscripts utilizing custom algorithms or software that are central to the research but not yet described in published literature, software must be made available to editors and reviewers. We strongly encourage code deposition in a community repository (e.g. GitHub). See the Nature Portfolio guidelines for submitting code & software for further information.

Data

Policy information about availability of data

All manuscripts must include a data availability statement. This statement should provide the following information, where applicable:

- Accession codes, unique identifiers, or web links for publicly available datasets
- A description of any restrictions on data availability
- For clinical datasets or third party data, please ensure that the statement adheres to our policy

Experimental and simulation data used in the present analyses are publicly available through Zenodo and University of York open access repositories, DOI:10.5281/zenodo.7786636 and DOI:10.15124/fd0eb563-9a9f-4c0d-82dc-27a4bf071660 respectively.

Research involving human participants, their data, or biological material

Policy information about studies with human participants or human data. See also policy information about sex, gender (identity/presentation), and sexual orientation and race, ethnicity and racism.

Reporting on sex and gender	N/A
Reporting on race, ethnicity, or other socially relevant groupings	N/A
Population characteristics	N/A
Recruitment	N/A
Ethics oversight	N/A

Note that full information on the approval of the study protocol must also be provided in the manuscript.

Field-specific reporting

Please select the one below that is the best fit for your research. If you are not sure, read the appropriate sections before making your selection.

- [x] Life sciences
- [ ] Behavioural & social sciences
- [ ] Ecological, evolutionary & environmental sciences

For a reference copy of the document with all sections, see nature.com/documents/nr-reporting-summary-flat.pdf

Life sciences study design

All studies must disclose on these points even when the disclosure is negative.

Sample size	In instances of reporting the development of the new COMBI-Tweez instrumentation in this manuscript, no formal sample size calculation was performed - in this manuscript we are primarily showcasing the capabilities of a new technology as opposed to obtaining extensive life sciences data. In instances where life sciences data was reported which involved reporting on the distribution (e.g.contour and persistence lengths of DNA tethers as detailed in Supplementary Fig. 13) we performed preliminary sampling to assess the s.d. width of the distribution and then determined heuristically the minimum sample size n such that the estimated s.e.m. would be at least 5 times smaller than the s.d.
Data exclusions	No data were excluded from the analyses.
Replication	In measuring the persistence and contour lengths of unlabeled and labeled DNA tethers replicates of 9 and 19 respectively were used. These replicates, produced values that converged on those expected when considering the theoretical values of contour and persistence length and on experimentally observed increases in persistence length when labeling DNA with intercalating dyes such as SYBR Gold.
Randomization	Our study was randomized since all tethered microbeads found and subsequently analysed went through attempted tethering and interrogation, with no user bias.
Blinding	Blinding in this study was unnecessary as the outcomes are not subjective but explicitly quantitative and are only specifically measured characteristics.

Reporting for specific materials, systems and methods

We require information from authors about some types of materials, experimental systems and methods used in many studies. Here, indicate whether each material, system or method listed is relevant to your study. If you are not sure if a list item applies to your research, read the appropriate section before selecting a response.

Materials & experimental systems

n/a	Involved in the study	
[ ]	[x]	Antibodies
[x]	[ ]	Eukaryotic cell lines
[x]	[ ]	Palaeontology and archaeology
[x]	[ ]	Animals and other organisms
[x]	[ ]	Clinical data
[x]	[ ]	Dual use research of concern
[x]	[ ]	Plants

Methods

n/a	Involved in the study	
[x]	[ ]	ChIP-seq
[x]	[ ]	Flow cytometry
[x]	[ ]	MRI-based neuroimaging

Antibodies

Antibodies used

Anti-Digoxigenin from sheep (Merck life Sciences 11333089001)

Validation

Supplier validation: "The polyclonal antibody from sheep is specific to digoxigenin and digoxin and shows no cross-reactivity with other steroids, such as human estrogens and androgens. Anti-Digoxigenin has been used in DNA tethering.[1] It has been used to attach DNA molecule to the glass surface of the flow cell.[2]"

1.) Methods in Molecular Biology (2011)

2.) Teeling-Smith, R.M. et al. Electron Paramagnetic Resonance of a Single NV Nanodiamond Attached to an Individual Biomolecule. Biophysical Journal (2016), 110(9), pp. 2044-2052
