# Supplementary material for: Correlating fluorescence microscopy, optical and magnetic tweezers to study single chiral biopolymers such as DNA
Source: Nat Commun. 2024 Mar 29;15:2748. doi: 10.1038/s41467-024-47126-6 (PMC10980717; doi:10.1038/s41467-024-47126-6)
Supplement: Supplementary file 19 — Description of Additional Supplementary Files [file 41467_2024_47126_MOESM19_ESM.pdf]

Supplementary Movie 1

Schematic video of the COMBI-Tweez system showing geometries alongside simulated and real data.

Supplementary Movie 2

Representative force extension experiment as performed in brightfield, see also Fig. 3. The maximum extension is set so that the optically trapped bead is just being pulled from the trap.

Supplementary Movie 3

An optically trapped bead tethered to an anchor bead is rotated, building up supercoiling density, forming plectonemes, and generating force. At a critical moment, the DNA buckles and the bead is pulled from the trap entirely. See also Fig. 3.

Supplementary Movie 4

DNA supercoiling with the force clamp applied. Here to keep the force constant the nanostage moves, reducing the distance between the anchor bead and optically trapped bead. See also Fig. 3.

Supplementary Movie 5

An anchor bead with single DNA molecules bound to the surface and imaged with SYBR Gold. To form a tether, a DNA molecule would be selected and the optically trapped bead brought into close proximity with it. See also Fig. 4.

Supplementary Movie 6

A force extension experiment performed during fluorescence imaging. See also Supplementary Movie 2 and Fig. 5.

Supplementary Movie 7

Two tethers between the anchor and trapped bead can be braided together by applying rotation to the optically trapped bead, increasing the braided region in the centre. See also Fig. 4.

Supplementary Movie 8

Two DNA molecules which are braided together are imaged, with one molecule breaking due to reactive oxygen species damage. The broken molecule retracts along the remaining tether. See also Fig. 4.

Supplementary Movie 9

A DNA tether imaged with SYBR Gold is seen breaking and retracting to the anchor bead due to entropic forces. See also Fig. 4.

Supplementary Movie 10

Video of a plectoneme formed by positive supercoiling. See also Fig. 5.

Supplementary Movie 11

Video of a DNA molecule buckling and pulling the optically trapped bead from the trap due to positive supercoiling.

Supplementary Movie 12

Video of a tethered full lambda DNA molecule following twisting show three plectonemes, which disappear sequentially following a single-strand DNA nick event followed by a torsional relaxation wave.

Supplementary Movie 13

MD simulation of the 362bp duplex at a supercoiling density of $\sigma = +0.1$ with a pulling force of 0.3 pN. See also Fig. 6.

Supplementary Movie 14

MD simulation of the 362bp duplex at a supercoiling density of $\sigma = +0.1$ with a pulling force of 0.7 pN. See also Fig. 6.

Supplementary Movie 15

MD simulation of the 362bp duplex at a supercoiling density of $\sigma = -0.1$ with a pulling force of 0.3 pN. See also Fig. 6.

Supplementary Movie 16

MD simulation of the 362bp duplex at a supercoiling density of $\sigma = -0.1$ with a pulling force of 0.7 pN. See also Fig. 6.
